# Genomes shed light on the secret life of *Candida glabrata*: not so asexual, not so commensal

**DOI:** 10.1007/s00294-018-0867-z

**Published:** 2018-07-19

**Authors:** Toni Gabaldón, Cécile Fairhead

**Affiliations:** 1 Centre for Genomic Regulation (CRG), The Barcelona Institute of Science and Technology, Dr. Aiguader 88, 08003 Barcelona, Spain; 2 Universitat Pompeu Fabra (UPF), 08003 Barcelona, Spain; 3 ICREA, Pg. Lluís Companys 23, 08010 Barcelona, Spain; 4 GQE-Le Moulon, INRA-Université Paris-Sud-CNRS-AgroParisTech, 91400 Orsay, France

**Keywords:** *Candida glabrata*, *Candida*, Fungal pathogens, Candidiasis, Genome sequencing

## Abstract

*Candida glabrata* is an opportunistic yeast pathogen, whose incidence has increased over the last decades. Despite its genus name, this species is actually more closely related to the budding yeast *Saccharomyces cerevisiae* than to other *Candida* pathogens, such as *Candida albicans*. Hence, *C. glabrata* and *C. albicans* must have acquired the ability to infect humans independently, which is reflected in the use of different mechanism for virulence, and survival in the host. Yet, research on *C. glabrata* suffers from assumptions carried over from the more studied *C. albicans*. Regarding the adaptation of *C. glabrata* to the human host, the prejudice was that, just as *C. albicans, C. glabrata is* a natural human commensal that turns deadly when immune defenses weaken. It was also considered asexual, as no one has observed mating, diploids, or spores, despite great efforts. However, the recent analysis of whole genomes from globally distributed *C. glabrata* isolates have shaken these assumptions. *C. glabrata* seems to be only secondarily associated to humans, as indicated by a lack of co-evolution with its host, and genomic footprints of recombination shows compelling evidence that this yeast is able to have sex. Here, we discuss the implications of this and other recent findings and highlight the new questions opened by this change in paradigm.

## *Candida glabrata*: the “other” *Candida*

*Candida* species are opportunistic yeast pathogens of increasing medical concern, as their infections can have high mortality rates, particularly when affecting immunocompromised patients (Angoulvant et al. 2016). The list of *Candida* species reported as etiological agents in human infections comprises up to 30 different species, and keeps growing almost every year with the inclusion of new rare species (Gabaldón et al. 2016). In this infamous hall of fame, *C. albicans* and *C. glabrata* are the first two ranking species based on their relative incidence (Angoulvant et al. 2016). *C. albicans*, accounting for over 40% of the infections, is the most studied species and has become a model for understanding *Candida* infections. As a result, knowledge acquired from the study of *C. albicans* is often taken as a framework to understand the other *Candida* species. However, *Candida* is not a true genus in the phylogenetic sense, as it comprises species that belong to different clades within the Saccharomycotina (Gabaldón et al. 2016). In particular, *C. glabrata* is only distantly related to *C. albicans*, being evolutionarily more related to the baker’s yeast *Saccharomyces cerevisiae* (Gabaldón and Carreté 2016). Hence, there is an inherent risk in adopting the knowledge acquired from *C. albicans* and assume everything works the same way in *C. glabrata*. Even if *S. cerevisiae* is taken as a closer model to understand *C. glabrata* (Roetzer et al. 2011), we must not forget that these two yeasts are still as far away from each other as we are from zebrafish in terms of sequence identity between orthologous genes.

*Candida glabrata* was first identified in human stools about a century ago by Anderson, which named it *Cryptococcus glabratus* (Anderson 1917). However, it only received significant attention when it started to be identified as the causative agent of fungal infections in immunocompromised patients (Just et al. 1989). In recent years, the incidence of *C. glabrata* has been increasing, perhaps due to its inherent higher resistance to commonly used antifungal drugs such as azoles (Angoulvant et al. 2016), and it has been referred to as an emerging pathogen since the late twentieth century (Hazen 1995). The first molecular studies performed on *C. glabrata* already pointed out a closer relativeness to *S. cerevisiae* than to *C. albicans* (Kurtzman and Robnett 2003). Soon, it was proposed to belong to the *Nakaseomyces* clade (Kurtzman and Robnett 2003), which comprises two additional pathogenic *Candida* species; *C. nivarensis* and *C. bracarensis*, and three environmental species. In 2004, the full-genomic sequence of *C. glabrata* was obtained (Dujon et al. 2004), and almost a decade later the sequences from all described species in the *Nakaseomyces* were completed and compared (Gabaldón et al. 2013). These comparisons provided unexpected insights on the emergence of virulence within the *Nakaseomyces* clade. First of all, the three species with virulence potential against humans were found to be non-monophyletic; suggesting that the ability to infect humans has emerged at least twice within this clade. Second, most of the traits once considered to be explanatory for the high pathogenicity (or frequent pathogenesis) of *C. glabrata* as compared to *S. cerevisiae*; such as growth at high temperatures or loss of the ability to synthesize nicotinic acid, were found to be widespread in the clade pointing to an ancient origin and, hence, disconnected from the origin of virulence. In contrast, expansion of cell wall adhesins, particularly those of the *EPA* family, were found to correlate with the virulent trait, suggesting that a potential for higher—or more versatile—adherence has facilitated the emergence of virulence towards humans among the *Nakaseomyces*. These results showed that *C. glabrata*’s ability to infect humans has evolved quite recently in evolutionary terms. Nonetheless, many questions remain open, particularly regarding the true lifestyle of this yeast.

## A secret life elsewhere, or a nomadic lifestyle?

How homogeneous is *C. glabrata* as a species? How does the genetic diversity of *C. glabrata* relate to the diversity of human populations? Are humans the main niche of *C. glabrata* or are we being colonized from our close environment? These are relevant questions for any pathogen, as resolving them may help understanding the epidemiology of infections and improve our chances to efficiently combat them. Different types of associations between a pathogen (or commensal) and its host exert different constraints on their evolution, which can be reflected in the genome sequence of the pathogen. For instance, the close association between the obligate pathogen *Mycobacterium tuberculosis* and humans, is reflected in strong parallelisms between the geographical projection of maps of genetic variation of both species (Comas et al. 2013). Studies on natural variation across *C. glabrata* isolates, have long been restricted to the analyses of a few loci or the measurement of few phenotypes (Dodgson et al. 2003, 2005; Brisse et al. 2009; Rolland et al. 2010; Schwarzmüller et al. 2014; Klotz et al. 2016). These studies detected the presence of several genetically distinct clades in the population of *C. glabrata* but the number of clades varied depending on the samples and markers used (de Meeûs et al. 2002; Dodgson et al. 2003, 2005; Brisse et al. 2009). These studies, which were based on a few loci, reported a significant enrichment in most of the clades in a particular geographical origin, suggesting that the distribution in *C. glabrata* was geographically structured and their population structure was predominantly clonal. Similarly, for *C. albicans*, an association between clades and the geographic origin is observed (Tavanti et al. 2005) and its mode of reproduction is described as predominantly clonal with limited recombination (Pujol et al. 1993; Fundyga et al. 2002). However, the low resolution imposed with the use of a limited number of loci restricted the conclusions that could be drawn from these studies. Overall, *C. glabrata* was generally considered a commensal of humans, although prevalence of *C. glabrata* in different mucosa vary from study to study, and generally increases with age and length of stays in hospitals (Angoulvant et al. 2016).

Nevertheless, *C. glabrata* strains have also been isolated from non-human sources, including from fermenting coffee beans (de Melo Pereira et al. 2014), and, most commonly, from droppings or cloaca swabs of several bird species (Cafarchia et al. 2008; Francesca et al. 2014; Al-Yasiri et al. 2016). Furthermore, *C. glabrata* can also be commonly found on the surface of hands or mobile phones of clinical workers (Kordecka et al. 2016). The central question here is whether isolation from a source supports the conclusion that the given source is a natural niche for the isolated species. Several lines of thought argue against this idea. First of all, the diversity of niches from which *C. glabrata* is isolated and the lack of common physiologically relevant characteristics among these sources (i.e., bird cloaca, mobile phones, and coffee beans) suggest that *C. glabrata* may just be a passenger in at least some of these sources—if not in all. This is consistent with the finding that *C. glabrata* is only sporadically—i.e., not systematically—isolated from these sources and in small amounts. Similarly, this is true for many other yeast species, including several other *Candida* such as *C. albicans*, that are found in the very same sources. For instance a recent survey have isolated three diverse strains *C. albicans* from oak trees, each one being closely related to a different clade of clinical isolates (Bensasson et al. 2018). Contamination from human sources could be a plausible explanation for the presence of such isolates or, alternatively, trees—or animals living on them—could be a natural reservoir for *C. albicans*. In any case, the example serves to illustrate the fact that isolation does not equate identification of a natural niche.

Many fungi tend to have a nearly ubiquitous presence and is difficult to identify their natural niche. Take, for instance, the well-studied *S. cerevisiae*. Although it is often purported as highly adapted to exploit fruits with high sugar content, it is only present in minute amounts on fruits and it is also commonly found—often at higher densities—in other environments such as tree barks, soils or humans. The ecological niche concept predicts that organisms should be more abundant in niches to which they are adapted to. However, alternative models in which organisms are highly versatile and able to survive across a broad spectrum of niches may better explain the ubiquitousness of certain microbes such as *S. cerevisiae* (Goddard and Greig 2015). Similarly, a “nomadic” lifestyle could explain the presence of *C. glabrata* in a diversity of niches, including humans. If this would be true, or if *C. glabrata* has a different natural niche, we should abandon the idea of *C. glabrata* as a highly specialized commensal but see it rather as an opportunistic colonizer. As we will see below, recent genomic comparisons seem to point to the idea that humans are indeed only a secondary niche of *C. glabrata*.

## No sex, or just hidden sex?

Another long-standing mystery surrounding *C. glabrata* relates to its ability to undergo a sexual cycle. Yeast species of the branch to which *C. glabrata* and *S. cerevisiae* belong, usually exhibit two mating types in haploid cells, which can fuse with each other to form diploid cells, with the potential to undergo meiosis and form spores (Muller et al. 2008). In addition, these yeasts are able to switch mating types, in a complex process that involves an endonuclease-driven cut, followed by recombination with silent loci, leading to replacement of transcription factor-encoding genes at the mating type locus (Haber 2012). Ploidy control and permanent availability to form diploid cells that can sporulate, may be the reason for the existence of such a complex phenomenon. Indeed, spores are the stage where cells can resist harsh conditions until the environment is more favorable to growth (Huang and Hull 2017). *C. glabrata*, like many other fungal pathogens of humans, is considered an asexual species and all attempts to make it mate in the laboratory have, so far, failed. However, the analysis of its genome sequence revealed the presence of homologs of all *S. cerevisiae* genes involved in the mating process (Fabre et al. 2005), and population analyses revealed the presence of cells of both mating types (Srikantha et al. 2003; Brisse et al. 2009). Moreover, the fact that *C. glabrata* cells of opposite mating types maintain distinct cellular identities (Muller et al. 2008), and evidence for mating-type switching in populations (Brisse et al. 2009), along with possible sexual recombination (Dodgson et al. 2005), suggest that *C. glabrata* may mate in yet undiscovered conditions with a canonical (i.e., *cerevisiae*-like), but rare, sexual cycle, or with a parasexual cycle, similar to *C. albicans*.

## Resolving puzzles from genomics footprints

**Fig. 1:**
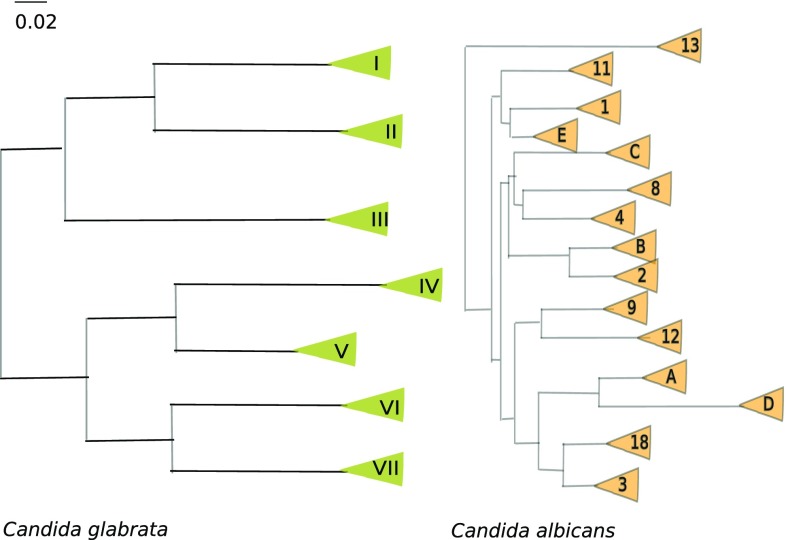
Molecular phylogenies of major clades *C. glabrata* (left) and *C. albicans* (right), as reconstructed from genome-wide single-nucleotide polymorphisms in recent studies (Carreté et al. 2018; Ropars et al. 2018). For comparison, both phylogenies have been drawn to scale in terms of average substitutions per site estimated in each of the studies

To gain novel insight into these open questions, we recently sequenced the genomes of 33 clinical *C. glabrata* isolates from around the globe and reconstructed their recent evolution (Carreté et al. 2018). Using this genetic information, we reconstructed a ‘family tree’ for all the strains. To our surprise, the genetic diversity of the species was structured in seven subpopulations (or clades) that had diverged deeply in the past (Fig. 1). For comparison, the genetic diversity found among distantly related clades was up to one order of magnitude higher than that found in worldwide sampled *C. albicans* isolates, and some of the identified clades comprised a similar level of diversity as that found between divergent *C. albicans* isolates. This means that clinical isolates of *C. glabrata show* more genetic diversity than that of *C. albicans* and, moreover, that this diversity is not homogenous but organized in a set of diverse genetic clades. Abusing of the—always conflictive in microbes—“species” concept to illustrate this, one could be talking about distinct *C. glabrata* subspecies, with some of these being as genetically diverse as the whole *C. albicans* species. This diversity was not only evident by the amount of sequence divergence, but also in a high level of variation in copy numbers resulting from gene deletions and duplications, which were enriched in regions coding for the abovementioned *EPA* adhesins and other cell wall proteins. To add to the colorful genetic diversity picture, we also identified several large genomic re-arrangements, including large inversions, translocations, and aneuploidies. Altogether, our genome comparisons revealed a highly plastic genome. We also measured some phenotypes of the sequenced strains and found a very high level of phenotypic variation, with important differences even between strains belonging to the same clade. This puts in perspective everything we “know” about *C. glabrata*, as most of the experimental knowledge from this species results from the analysis of one or two “model” lab strains.

Perhaps the most important observation, however, was that there was no apparent geographical structure in this genetic diversity. On the contrary, each clade seem to contain strains isolated from distant locations such as Europe and America. This is at odds with the idea of co-evolution between humans and *C. glabrata* as it would be expected for a tight commensal relationship. Rather, it suggests that previously isolated populations have recently inter-mingled, as if some new factor has favored this trend. Global spread of species is often related to human-related phenomena, such as global trade and climate change, and it is very likely that human action is behind the recent global spread of these clades. However, the long branches separating the different clades are telltale signs of long isolation, likely in distinct geographical areas. Thus, the genetic diversity and geographical dispersion of genetic backgrounds seem to strengthen the idea that humans are only a secondary niche for *C. glabrata*, and that we have facilitated its global expansion. A high versatility and a possibly nomadic lifestyle has likely favored the expansion of *C. glabrata* and would also explain the ease with which it can colonize us, and cause severe infections when our defenses are weakened.

The existence of clearly genetically differentiated clades gave us another useful opportunity: find the smoking gun of past sexual reproduction in the form of genomic recombination. Sex between cells results in genetic exchanges between the partner genomes. If this has happened between cells of different clades—which we now know co-exist globally—this would result in chimeric patterns of genetic diversity along the chromosomes of the descendants, just like the chromosomes that we transmit through our gametes consist of stretches of genes coming from our mother and father. This is exactly what we found in some of the sequenced strains, suggesting the different clades have exchanged genetic material and providing a compelling evidence for this species’ ability to have sex. In accordance with this, we found evidence for high levels of purifying selection in the genetic toolkit required to have sex, indicating that deleterious mutations in these are removed by natural selection in *C. glabrata* populations. Furthermore, our analysis has confirmed the existence of mating-type switching, and of illegal repair events during switching in the population. Experimental results show that switching is lethal to many *C. glabrata* cells in laboratory conditions (Boisnard et al. 2015). Although sex can occur in non-switching species, it is conversely admitted that switching exists to favor sexual reproduction. The conservation of this complex and dangerous mechanism in *C. glabrata* is another indication of the existence of cryptic sex. Thus, this yeast is not asexual, we just do not know what triggers its sexual cycle.

## Future perspectives

What are the implications of these recent discoveries enabled by reading the genomes of a collection of clinical strains? We believe they are many. In the first place, they have uncovered an astonishingly high genetic diversity in this pathogenic yeast. In addition, the *C. glabrata* genome seems to be very dynamic, and can be easily shaped by gene gain, loss and large genomic re-arrangements. Thus, research on the current single “lab strain” is of limited use, unless it is complemented with some validation in strains from different clades. Second, an unexpected lifestyle seems to be implied from this results: *C. glabrata* is probably environmental and sexual, which has deep implications in how we should understand its relationships with the human host and its epidemiology. We may be facing a menace that is coming from an unknown environment. This menace is highly diverse and plastic, and able to have sex. This sexual ability may favor the exchange of genetic material, thereby accelerating its adaptation. A worrying hypothetical scenario is the possibility of a drug resistance expanding through genetic exchange across populations.

Current studies, some of which performed by us, are expanding the breath of the genomic comparisons, focusing on groups of isolates showing phenotypes of interest, such as those related to virulence, drug resistance, and adhesion. A final goal of these analyses is to uncover genetic factors determining these traits, that is, establishing links between phenotype and genotype. Moreover, other studies may focus on the comparison of serial isolates taken from the same patient at different times in the course of an infection. The idea is to understand microevolutionary processes leading to the emergence of drug resistance—or persistence (Bojsen et al. 2017)—or a more invasive phenotype. Last, but not least, the environment should be tested for the presence of *C. glabrata* with modern identification techniques. Isolating a given species in the many ecosystems that exist around humans may be like finding a needle in a haystack; but, until this is done in an extensive manner, the jury will remain out on the question of the environmental reservoir.
